# Teach to Beat Cancer: An Integral Component of the Case Comprehensive Cancer Center Youth Enjoy Science Program

**DOI:** 10.15695/jstem/v5i2.05

**Published:** 2022-08-03

**Authors:** Damian J. Junk, Nathan A. Berger

**Affiliations:** 1 Case Comprehensive Cancer Center, Health, and Society, Case Western Reserve University, Cleveland, OH, USA; 2 Center for Science, Health, and Society, Case Western Reserve University, Cleveland, OH, USA

**Keywords:** Teachers, Cancer Education, Training, Youth Enjoy Science Program

## Abstract

The Youth Enjoy Science -Teach To Beat Cancer program funded by the National Cancer Institute utilizes the resources of the Case Western Reserve University School of Medicine and Case Comprehensive Cancer Center to provide experiences and training for grade 6-12 teachers so that they are expertly equipped to develop curricular approaches to take back to their classrooms to enhance science education, introduce concepts of cancer biology, encourage cancer risk reduction activities, foster disparity elimination and to motivate middle and high school students to pursue careers in biomedical sciences and cancer research. The program focuses on four aspects of teacher engagement and education: 1) Research Engagement, 2) Curriculum Development, 3) Risk Reduction and Disparity Elimination, and 4) Advocacy for Cancer Research and Cancer Research Careers. This program has been crucial to achieve the National Cancer Institute’s goal of educating students from diverse backgrounds underrepresented in biomedical research in the Cleveland area as each teacher influences a significant number of students in their classrooms each year, and are introducing students to cancer biology, exciting them to consider careers in cancer prevention, diagnosis, control, treatment, and research. This article provides an overview of the program including its impact on the teachers and their students.

## INTRODUCTION

Racial and ethnic minorities are significantly underrepresented in the science, technology, engineering and math (STEM) professions, with the composition of the life science workforce being 67% White, 19% Asian, 4% Black, and 7% Hispanic (Funk and Parker, 2018). Likewise, the cancer research workforce has significant underrepresentation of minority group members, although these groups are among the most rapidly growing and suffer the greatest burden of cancer (AACR 2020). Efforts to improve the participation of minorities in the cancer research and care professions are an important national priority with the expectation that increasing diversity of the cancer research and care workforce will contribute to reducing the burden of cancer in minority populations, eliminating cancer health disparities and achieving cancer health equity (Carpten et al., 2021).

The National Cancer Institute (NCI) has instituted multiple programs, across the education spectrum, to increase diversity among cancer research and healthcare professionals (National Cancer Institute www.extramural-diversity-NIH.gov/IG-pages/national-cancer-institute), including the R25 Youth Enjoy Science (YES) Program, which focuses on engaging middle school, high school and undergraduate students, as well as science teachers to expand the pipeline of URM students entering cancer research. This report focuses on the teacher component of the program at Case Western Reserve University (CWRU) and the Case Comprehensive Cancer Center (Case CCC) located in Cleveland, Ohio.

The population of Cleveland is 376,549 persons, with a racial/ethnic composition that is 50% Black, 34% White and 12% Hispanic (CMSD www.clevelandmetroschools.org/domain/24). With a median household income of $30,903 and a poverty level of 32.6%, Cleveland is one of the poorest large cities in the US where 42% of children and 52% of children under the age of five live below the poverty level. The Cleveland Metropolitan School District (CMSD), with 37,158 students has a racial ethnic distribution of 64% Black, 15% White, 16.7% Hispanic and 4.2% other (CMSD www.clevelandmetroschools.org/domain/24). Through a variety of programs and fundings, the high school graduation rate of CMSD has improved from 26% in 2010 to 72.1% in 2021 (CMSD www.clevelandmetroschools.org/domain/24). Among new 2020 high school graduates, 36% matriculate to college (O’Donnell 2021).

**Figure 1.**
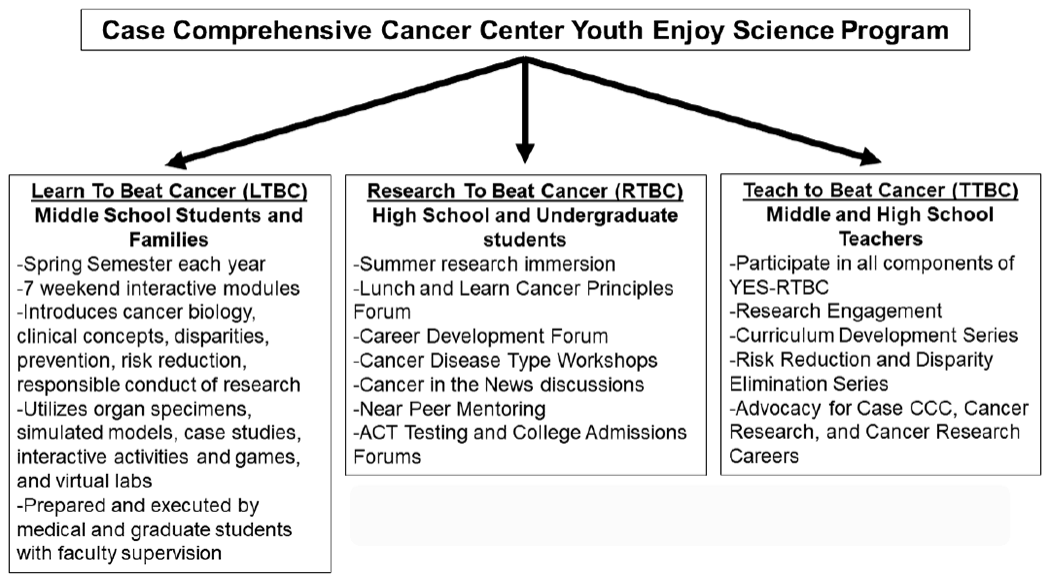
The Case CCC YES Program: The YES program consists of three integrated components targeted to specific participant populations.

The CWRU SOM and Case CCC coordinate the highly integrated Scientific Enrichment Opportunity (SEO) and Youth Enjoy Science (YES) STEM programs (SEO/YES), to engage local Cleveland-area students in immersive biomedical cancer-focused research and education, and to encourage their pursuit of careers in oncology-related research and clinical care (Qua et al., 2020a; Qua et al., 2020b; Qua et al., 2021). The YES component of the SEO/YES program, now entering its fifth year, is supported in part by an R25 training grant from the NCI to facilitate the education of students from diverse backgrounds underrepresented in biomedical sciences who will become knowledgeable about cancer, and focus on cancer related professions later in their careers. The SEO component, supported by philanthropy, has broader community representation. The combined components are delivered as the SEO/YES program. The SEO/YES program has shown the importance of providing STEM education and research experiences (Qua et al., 2020a; Qua et al., 2021) as well as near peer and faculty role models (Qua et al., 2020b) to science-motivated students underrepresented in biomedical sciences. Since its inception, the Case CCC YES program has been divided into three major components: 1) Learn To Beat Cancer (LTBC), focused on middle school students with outreach to their families, 2) Research To Beat Cancer (RTBC), focused on high school and undergraduate students, and 3) Teach To Beat Cancer (TTBC), focused on middle and high school science teachers. The three components and their associated activities are shown in Figure 1. These three unique components are integrated in content and delivery to constitute the overall SEO/YES program at the Case CCC.

**YES-LTBC** provides an intervention at an early age to engage, excite, educate, and encourage students underrepresented in biomedical science from grades 6, 7, and 8, and their family members in principles of cancer biology, clinical concepts, disparities, prevention, risk reduction, and responsible conduct of research to motivate them to pursue careers in health care and cancer research. All YES-LTBC workshops are designed to emphasize active and hands-on participatory learning employing interactive team inquiry methods.

**YES-RTBC** is designed to engage the scientific curiosity and promote the potential cancer -focused careers of promising high school and undergraduate students underrepresented in biomedical research. YES-RTBC is an eight-week summer program focused on rapidly immersing each student in an individual mentored cancer-research project utilizing Case CCC and CWRU SOM faculty and facilities. Student projects are aligned with student personal interests. In addition, YES-RTBC provides active teaching didactic curriculum including weekly Lunch and Learn Cancer Principles and Career Development Forums, Cancer Disease Type Workshops and student-led discussions of Cancer in the News (Qua et al., 2021).

**YES-TTBC**, the focus of this manuscript, provides grade 6-12 teachers with opportunities to engage in scientific research and education that will enhance their skills, knowledge and abilities to teach science and more specifically to focus on principles of cancer biology. YES-TTBC teachers participate alongside high school and undergraduate students in all aspects of the YES-RTBC component (cancer-research immersion, Lunch and Learn Cancer Principles Forum, Career Development Forum, Cancer Disease Type Workshop, Cancer in the News, and Near Peer Mentoring). In addition, all teachers meet weekly as a group for unique teacher-focused workshops to develop cancer-focused curricula to take back to their students, classrooms, and schools.

## YES-TTBC DESCRIPTION

The goal of YES-TTBC is to use the resources of the CWRU SOM and Case CCC to provide experiences and training for grade 6-12 teachers so that they are expertly equipped to develop curricular approaches to take back to their classrooms to enhance science education, introduce concepts of cancer biology, encourage cancer risk reduction activities, foster disparity elimination and to partner with the Case CCC to motivate middle and high school students to pursue careers in biomedical sciences and cancer research. YES-TTBC focuses on four aspects of teacher engagement and education: 1) Research Engagement, 2) Curriculum Development, 3) Risk Reduction and Disparity Elimination, and 4) Advocacy for the Case CCC, Cancer Research and Cancer Research Careers.

### Research Engagement.

The focal point of YES-TTBC is to provide grade 6-12 teachers with a cancer-research project utilizing Case CCC and CWRU SOM faculty and facilities. Teachers are trained in the scientific method, responsible conduct of research, laboratory techniques, safety policies, and work with individual faculty mentors to develop an independent research project within the overall scope of the mentors’ laboratories. Teachers conduct hands-on, cancer-focused experiments and research projects during the eight-week summer research immersion with an optional four-week research extension. They present their findings at the end of the summer capstone SEO/YES Symposium. When feasible, teachers are paired with high school or undergraduate students for research immersion, to familiarize teachers with student research participation, to inform teachers of student excitement and challenges, and to assist teacher development of active curriculum design for students.

### Curriculum Development.

Teachers are guided in curriculum development by exposing them to research experiences and helping them to develop strategies, focusing on active learning styles (Ballen et al., 2017; Freeman et al., 2014; Theobald, 2020) to provide them with tools and guidance to excite and interest middle, high school, and undergraduate students to explore careers and opportunities in cancer research (Hurtado et al., 2009; Penuel et al., 2022). Importantly, active learning compared to traditional passive learning approaches has been shown to increase student performance in STEM subjects (Freeman et al., 2014). Teachers are expected to generate curricula each year to take back to their classrooms and incorporate into their yearly planning for their upcoming science courses. As each teacher leads different science courses for students at variable grade levels they develop curricula appropriate to student understanding and content based on the tenets of cancer biology, research, prevention, and treatment that are consistent with the courses they are leading. Teachers meet weekly as a group over the summer for unique curricula-focused workshops (Table 1).

### Risk Reduction and Disparity Elimination.

Teachers are provided with weekly interactive discussion seminars on cancer risk factors, strategies for risk reduction and disparity elimination, and encouraged to incorporate these strategies into education initiatives for students as well as for their families (Merten et al., 2017; Zeinomar et al., 2021). Principles learned include the cancer risk associated with obesity, diabetes, decreased exercise and physical activity, sleep deprivation, tobacco use, sexual activity and UV exposure. Teachers are instructed in development of strategies to teach cancer risk reduction focused on activities such as nutrition and weight control, elimination of tobacco use, increasing physical activity and exercising, improving sleep hygiene and use of HPV vaccine in both girls and boys. Teachers are encouraged to teach students and to further reach out to parents to promote risk reduction and to understand and utilize screening procedures for skin, breast, gastrointestinal, gynecological, and prostate cancers. Each year the teachers are invited to present and participate in the annual Case CCC Cancer Disparities Symposium, a national meeting of leading experts in the field of cancer disparities research, and cancer prevention diagnosis and treatment. Teachers develop in-class curricula to take back to their schools to foster awareness of cancer disparities, to have the students develop strategies for disparity reduction, and to highlight the importance of obtaining early and comprehensive care.

### Advocacy for Case CCC, Cancer Research, and Cancer Research Careers.

Teachers are introduced to components, programs, and faculty of the Case CCC to engage them as community ambassadors and advocates for student pursuit of careers in health sciences and cancer research. Teachers are provided with tours of the clinical and research facilities of the Case CCC and CWRU SOM. Moreover, they are introduced to clinical leaders, especially underrepresented in biomedical sciences physicians and researchers to encourage and enhance the teachers’ ability to become advocates for the Case CCC and to encourage community members to participate in clinical research. The clinicians and researchers underrepresented in biomedical sciences also provide role models the teachers can invoke when inspiring students to consider careers in cancer research, prevention, and treatment (Kricorian et al., 2020). Participation in YES-TTBC provides teachers with a greater understanding of the mission of the Case CCC and the National Cancer Institute and the desire to help their communities with cancer prevention, diagnosis, treatment, and clinical trial participation.

### Timeline.

Requests for application are distributed after the new year and applications are accepted during the month of February. After faculty review, teachers are selected for the summer program and notified during March. The eight-week TTBC program starts the first week of June with an orientation meeting for both students and teachers. During the first week, both teachers and students are provided with safety training, discussion of responsible conduct of research, introduced to faculty mentors and are immersed in individual laboratories for their research projects. Conduct of research and regular daily seminars are ongoing concurrently for the eight-week period culminating in the capstone presentation at end of the program in July. During the entire program, teachers meet weekly with program leadership for special learning sessions, and developing strategies for curricula development which they then continue as collaborative projects (Table 1). At the end of the formal, summer eight-week education and research program, teachers are eligible to participate for up to four additional weeks. Teachers may continue participation through attending weekly Case CCC Seminar Series, attending the Case CCC Annual Disparities Conference, continued development of classroom curricula with YES leadership, and/or an extension of laboratory research with their mentors with support from the YES program. After completing the first year of program training, returning teachers are invited to assist with high school student selection for program participation, to evaluate student presentations for merit awards at the end of summer capstone event, and to work with YES leadership to develop and implement YES activities for the next summer student immersion.

## RESULTS

### Teacher Recruitment, Selection, Compensation and Participation.

Teachers are recruited to the program by invitations and applications mailed to surrounding school district and high school leadership including superintendents, principals, and science education coordinators. Teachers are selected for program participation by a panel of CWRU SOM and Case CCC faculty members, based on their applications and letters of recommendation from school principals and/or department heads. Teachers are compensated for participation at the NCI-designated rate of $900 per week for their 8-12 week involvement (8 weeks for the required summer immersion and up to 4 weeks additional participation throughout the academic year).

Demographics for participating teachers and the schools at which they teach are included in Table 2. Over the past grant cycle (summer 2018, 2019, 2020, and 2021), the YES-TTBC program provided 19 summer training experiences for 10 individual teachers. The majority (9) are teachers of science (biology, chemistry, physics, environmental and life sciences) courses at the high school level, and one is a middle school science teacher. Most of the teachers (8) hold Master’s degrees, one holds a Bachelor’s degree, while another holds a PhD in Biochemistry. Participating teachers range in age from 29 to more than 65 years old. Eight of the teachers work within the CMSD and two work in Cleveland suburban school districts. Of the 10 teachers in the program, three have participated for three years, three for two years, and four for one year (Table 3). Three teachers who received their first year of support during the summer of 2021 have already applied for the program in 2022 (Table 3). At this time, one teacher has not continued participation for a second year due to personal issues unrelated to the program. As shown in Table 3, four to six teachers participated as a cohort each year, and the number of teachers supported has gradually increased over the grant cycle (Table 3).

### Teacher Research.

All ten teachers conducted a summer immersion research project with a Case CCC mentor each year, presenting their research at the annual capstone end of program research symposium. Some of the projects included: “Characterization of Novel Small-Molecule Inhibitors of a Human Base Excision Repair Enzyme, Uracil DNA Glycosylase”; “Study of the Effects of Dendritic Cell Treatment in Relation to Autoimmune Diseases”; “Isolating and Identifying Genes that Contribute to Breast Cancer Dormancy”; and “Dual Contrast Magnetic Resonance Fingerprinting Assessment of Water Mobility for Cancer Imaging” among others. During pandemic imposed remote contact years, teacher research focused on team-based efforts to explore pedagogical approaches for curriculum development. In addition, several teachers have presented posters at the annual Case CCC Disparities Symposium, including “Teaching to Beat Cancer: Expanding Genetics and Cancer Awareness in Urban High Schools.”

### Curricula Generation.

Teachers generated curricula each year (8 total topics to date) to take back to their classrooms and incorporate into their yearly planning for upcoming science courses. Table 4 provides a list of pedagogical approaches to active learning, introduced by participating teachers. Their uses are described below in relevant teaching modules. It is important to note that multiple approaches can be utilized in each module. For example, a case-based study can be used to engage student interest because of its personal connection, then inquiry-based learning can be conducted at the individual or team level to identify subject matter to be mastered.

As each teacher leads individual science courses for students at different grades, they develop curricula appropriate to the level of student understanding and content based on the tenets of cancer biology, research, prevention, and treatment that is consistent with the courses they are leading. Here we provide brief examples of the curricula developed and implemented in the YES TTBC Program.

1) A module titled “A Cancer Story” was developed as a unit for the introduction of biotechnology techniques focused on the customized treatment of cancer for AP Biology and Biotechnology/Genetics courses. The module utilizes the “Faces of Cancer” first developed at the National Cancer Institute as physician case studies for the students to play the role of treating physician. (National Cancer Institute Research Advocacy 101: Human Faces of Cancer at NCI) Students are challenged at the end of the module to integrate what they have learned about the cell cycle, DNA sequencing, and biotechnology techniques to develop a personalized treatment plan for a fictitious cancer patient.

2) A module entitled “How to Beat Cancer” was developed for a 9th grade science course. The weeklong module includes a personal introduction to an anonymous mother that is diagnosed with cancer. The students are walked through a discussion of how the cancer affects the entire family to bring it to their level as they see through the eyes of the patient’s children. Students are then asked about cancer facts and myths, followed by an introduction to basic cancer biology. Students are then introduced to careers in oncology and cancer research while learning cancer treatment techniques utilized or developed by each career choice. Finally, students are assigned a project to develop strategies they can use to lower their own lifestyle-behavioral risk of developing cancer, for example smoking cessation, exercise, diet, and HPV vaccination.

3) A module entitled “Disparities in Cancer and Healthcare” was developed by a group of three teachers. The group worked to define cancer, identify data that demonstrates population differences in incidence and response to treatment, define cancer and health disparities, identify factors that lead to disparities, and understand potential solutions. Then each member used the group work to develop a classroom intervention specific to their science course and academic level of students. In each teacher’s curriculum, classroom discussions were built into the module so that students could discuss their own situations (neighborhoods, diets, family history, knowledge of healthcare) and develop their own potential solutions to cancer and healthcare disparities for their communities and beyond.

4) A module on “Hereditary Cancers” focused on a young woman with Hereditary Non-Polyposis Colorectal Cancer (HNPCC-Lynch Syndrome). This module provided the basis to discuss colorectal cancer, genetics, development, screening and therapeutic options. In preparation for development of this module, teachers met with genetic counselor faculty who instructed them in the construction and interpretation of family pedigrees, which were then used by teachers in their classrooms to have students analyze hereditary cancers as well as other hereditary diseases such as sickle cell disease and hemophilia A&B to provide examples of different patterns of genetic disease transmission. In addition to discussing the cancer specific science, this module provided the interesting opportunity to engage students in discussion of ethics of disease disclosure, especially to potential partners, as well as reproductive options to avoid disease transmission.

5) Students were introduced to a young patient of their own age who was diagnosed with “Acute Lymphoblastic Leukemia”. A case-based approach allowed students to identify with the patient of their own age. Using the scientific inquiry approach, students were engaged to obtain, evaluate and organize evidence to reach and communicate understanding of disease pathophysiology. The module was used to examine blood cell structure and function, cell division and cell cycle, and DNA structure, function and mutation.

6) In a module entitled, “Tobacco Use, Heart Disease and Cancer” the teacher presented his own history of heavy smoking, heart disease and stroke, as a basis to apply social-emotional learning to engage student empathy, to evaluate tobacco use as a risk factor for heart disease, lung, and bladder cancer, and to motivate students to develop positive life approaches for tobacco control.

7) Another case-based module focused on a family friend’s 14-year-old son diagnosed with “Glioblastoma” and being treated with a ketogenic diet. Using a problem-based approach, students learned about Hallmarks of Cancer, cancer staging, therapeutic options and adjuvant therapy. The ketogenic diet was further used to examine energy balance, carbohydrate and lipid metabolism used by normal and tumor tissues.

8) Development of a module focused on “Drug Development and the FDA Approval Process” was motivated by controversy surrounding safety and utilization of COVID-19 vaccines. The module provided students an opportunity to undertake guided inquiry to examine the drug-vaccine development and approval process, and to better understand preclinical testing, phase I, II, and III clinical trials, emergency use authorization, and FDA approval. In addition to better understanding the processes involved, the module prepared students to discuss issues of drug-vaccine safety with individuals who were COVID-19 vaccine resistant, as well as to become advocates for cancer clinical trials.

All of these modules have been presented by participating teachers as adapted to different grade classes. These approaches have not yet been quantitatively evaluated for subject matter mastery. However, in overall formative assessments, teachers report that students have enthusiastically engaged with the cancer focus and active learning approaches. Overall, teachers indicated that curricula modules with case-based presentations, that actively involved student participation, were most effective in engaging their interest, especially when the students could relate to the case on a personal level. Of equal importance for engaging students was providing them the opportunity to relate to their own situation of neighborhood, diet and/or family history.

### Evaluation.

Each year, YES program leadership evaluated the YES-TTBC component through informal exit interviews and program observation. Among many potential enhancements to the YES program overall and the YES-TTBC component specifically, six key observations were consistently noted (Table 5). Feedback from the teachers showed that they knew very little about the structure, purpose, and activity of the National Cancer Institute, CWRU or the Case CCC, nor did they know how to engage the Case CCC prior to joining YES-TTBC. For example, teachers were not aware of the NCI mission to lead, conduct, and support broad spectrum cancer research to advance scientific knowledge to help all people live longer, healthier lives (National Cancer Institute www.cancer.gov/aboutNCI). Of significant importance for their role in guiding students, teachers were unaware of the NCI commitment or programs to encourage individuals from diverse backgrounds, including those from underrepresented in biomedical and behavioral science, to pursue cancer research, to improve their career opportunities, to broaden the cancer research workforce and to contribute to elimination of cancer disparities. (National Cancer Institute www.extramural-diversity-NIH.gov/IG-pages/national-cancer-institute) Teachers were similarly unaware of Case CCC outreach programs. While YES-TTBC provided opportunities for the teachers to tour Case CCC facilities and to develop meaningful relationships with Case CCC leaders and members, only a small subset of facilities was included each year due to the short duration of the summer program.

Teachers that participated multiple years had the opportunity to explore more of the Case CCC and expand their networks with more of the Case CCC Faculty. In addition, teachers gain sufficient training and education to understand cancer and scientific research in the first year, but in subsequent years they gain greater facility and enhance their confidence with experimental techniques and are more capable of translating their new-found knowledge to effective curricula to take back to their classrooms. Additional training years often enhanced their breadth of knowledge as the teachers typically work in an alternate lab with a different cancer focus than their first year. Returning teachers from previous cohorts increased, not only, their own learning and cancer expertise, but also, assisted the training of the new teachers in each cohort.

As the program has grown over the grant cycle, we found that a cohort size of six teachers is optimal. This group size allows teachers to work in small groups on projects or to develop curricula of shared interest, while also allowing some teachers who prefer to do so, to work as individuals. This cohort size is also manageable for the program to make laboratory placements, provide successful training, allow tours of Case CCC facilities as a single group or two groups of three when space is limited in the facility, and allow effective discussions among the group giving each an opportunity to present their understanding of what they have learned and ask questions. Therefore, we target a teacher cohort of six individuals each year with 2-3 returning teachers and 3-4 new teachers.

We also found that the teachers prefer to work alongside students on the research projects during the summer laboratory immersion. This research project pairing, implemented when feasible, allowed teachers to appreciate research activities from the viewpoint of a high school or undergraduate learner, to guide students to secure maximum benefit from the experience, and to advise program leadership and research staff on approaches to improve the overall program for hosting students. The teachers enjoyed assisting student learning and growth while sharing the responsibility of learning new information and laboratory techniques, often learning a great deal from each other. When teacher/student pairs could be implemented, they were highly productive and provided the teachers with the student perspective of research to help inform their curriculum development. Therefore, we will continue to emphasize student-teacher pairing for all teachers during the laboratory research immersion in future years.

Teacher feedback also showed that they would appreciate outreach, such as HPV vaccination information, from the Case CCC to their schools and classrooms pertaining to cancer disparity, prevention and control. Often, the teachers who completed YES-TTBC the previous summer invited Case CCC faculty from their newly developed professional network from YES-TTBC participation to present to students in their classrooms. While YES program leadership understands the importance of having students attend our summer programming on the CWRU campus to help the students envision themselves as future college students at a premier research institute, we also realize the importance of meeting the students in their own space in order to excite them about future careers in biomedical sciences. Outreach to individual schools often increased awareness of the summer program among their schools and increased applications from visited schools in subsequent years. Therefore, we intend to increase our outreach to students and schools through our connections with YES-TTBC participant teachers.

Finally, as a result of their programmatic exposure, teachers actively sought increased roles in YES-TTBC and the Case CCC. As the SEO/YES program has evolved, we asked the teachers to lead two sessions of our YES-RTBC Cancer in the News weekly workshop. The teachers were responsible for identifying two related headlines of recent science discoveries in the news that might affect the students. The teachers distributed the news stories, provided a mini-lecture of background information pertaining to the development of the story and led student discussions of the scientific discovery and how it affects their lives and that of their peers and families. This activity allowed us to assist the teachers with researching the discovery, preparing the lecture for students, delivery of content, and to provide feedback of their methods and delivery techniques to encourage active styles. These activities significantly enhanced their cancer curriculum development by encouraging the teachers to utilize active learning techniques and engaging approaches to further excite their students about cancer research, prevention and treatment. The teachers enjoyed employing their own styles while learning new methods and being an integral part of the SEO/YES program. As the teachers began to feel a part of the Case CCC team they sought out additional opportunities to engage and advocate for the cancer center. Importantly, teachers from previous cohorts continue to attend the Case CCC Annual Disparities Conference (spring each year) after they have completed their YES-TTBC training. In addition, one teacher expressed a strong desire to work alongside the Case CCC, was introduced to our Office of Community Outreach and Engagement and is now an engaged advocate member of the Case CCC Community Advisory Board. Therefore, we will seek additional opportunities for teachers to develop, implement, and engage in student training activities of the SEO/YES program.

## DISCUSSION

YES-TTBC is a critical pillar of the Case CCC SEO/YES program supported by the NCI to facilitate the education of Cleveland area students from diverse backgrounds underrepresented in biomedical research who will become knowledgeable about cancer, and available to focus on cancer research and care later in their careers. Prior to participation in YES-TTBC, most Cleveland teachers have a limited understanding of the mission, goals, and resources of the NCI and of Case CCC. YES-TTBC provides opportunities for the teachers to develop meaningful relationships with Case CCC leaders and faculty, and opportunities for them to engage in Case CCC activities and advocacy. Accordingly, we consider it important to provide opportunities for teachers to participate in Case CCC seminars, conferences, and activities after they have finished the program to continue to foster their professional networks with faculty of the Case CCC and CWRU SOM.

Program evaluation showed that multiple years of participation in the YES-TTBC program by teachers is critical and that a cohort size of six teachers consisting of a mix of returning and new teachers is optimal. Returning teachers from previous cohorts increased, not only their own learning and cancer expertise, but also assisted the training of new teachers in each cohort. Three years of participation significantly enhanced the breadth of understanding of cancer, cancer research, and cancer curriculum development. In addition, having the teachers actively lead established components of the SEO/YES program (Cancer in the News) gave them practice teaching and allowed YES leadership to observe and provide feedback for researching, developing, and delivering cancer content to their student classrooms. Participating as leaders of SEO/YES program components and receiving feedback from leadership further encouraged them to utilize active learning techniques and engaging activities to excite students about cancer research, prevention and treatment. Thus, it is critical to select teachers that are able to commit to at least two and potentially three years of participation, incorporating them into the design and implementation of the student-focused activities of the SEO/YES program, and providing feedback from our expert faculty for their delivery of content. As noted above, all teachers have committed to two years at the time of application. In only one case this became impossible due to external, non-programmatic related circumstances.

Solicited feedback at the end of each year of the program demonstrated that the teachers preferred working alongside high school students during their laboratory immersion, and that teacher/student pairs were highly productive and provided the teachers with the student perspective of research to help inform their curriculum development. Future teacher applications to the YES program will continue to encourage teachers to identify and apply with a student from their classrooms to work as a teacher-student pair. Teachers who apply solely as individuals will be paired with students underrepresented in biomedical sciences from their school, if possible, or from another school, if necessary, for the research experience.

Teachers often sought outreach from the Case CCC to their schools and classrooms following participation in the program over the summer. Feedback from all teachers suggested they would appreciate opportunities to have program leadership and members of the Case CCC visit their schools and classrooms each year. SEO/YES program leadership is working with the Case CCC Office of Community Outreach and Engagement to formally provide outreach activities to each of the YES-TTBC teacher classrooms at least once a year while they are participating in the program. This will include opportunities for guest lectures from one of our exceptional faculty, HPV vaccination outreach, or screening and prevention outreach from our clinical faculty.

The teachers involved in YES-TTBC were very excited to participate in the program, but also sought opportunities to become active members in the enterprise of the Case CCC. The teachers enjoyed using their skill sets to design, implement, and execute the student-focused programming of the SEO/YES program. They also consistently sought opportunities to stay engaged with the Case CCC after completion of YES-TTBC, such as attendance each year at the Annual Case CCC Cancer Disparities Conference. As noted above, one teacher has now become an engaged advocate member of the Case CCC Community Advisory Board. The SEO/YES program will continue to identify opportunities for teacher engagement within the program and with the CWRU SOM and Case CCC at large.

The Case CCC YES program supported by the NCI has been crucial to achieve the goal of educating students from diverse backgrounds underrepresented in biomedical research in the Cleveland area. While the program provides annual funds to support the participation of a fixed number of Cleveland area high school students each summer, providing training for Cleveland school teachers significantly increases the overall Cleveland area student impact. Each teacher influences a much larger number of students in their classrooms each year, and they are introducing students to cancer biology and exciting them to consider careers in cancer prevention, diagnosis, control, treatment, and research. At the same time, the teachers are facilitating additional outreach to Cleveland area schools from members of the Case CCC and CWRU SOM.

## Figures and Tables

**Table 1.** YES-TTBC Workshop Activities.

**Week 1: Introduction**	Welcome and overview of the YES-TTBC Program. Explanation of the expectations of teachers to develop teaching plans, curriculum, and materials to take back to their respective schools. The focus for the teachers will be Cancer Prevention, and how to disseminate information and healthy lifestyle tips to their students.
**Week 2: Working meeting**	Discuss plans for curriculum development. Envision the classroom component of the work and devise a final presentation format. Determine what teachers are providing currently and how that offering can be expanded or enhanced with information gleaned from the summer program. Introduction to active learning and teaching techniques.
**Week 3: Colon Cancer Family Syndromes**	Presentation of Familial Adenomatous Polyposis and Lynch Syndrome Colon Cancers. Development of a colon cancer focused familial risk and prevention learning module for students.
**Week 4: Genetic Risks of Cancer**	Explanation of cancer as a genetic disease and the increased risk associated with family history. Teachers develop a family pedigree of cancer risk as a model for an activity to conduct with their students to help students and their families identify any increased cancer risks, and to talk to their families about cancer and the importance of understanding and sharing their medical histories.
**Week 5: Working Meeting**	Teachers were shown a variety of DNA models to explain mutation leading to cancer. Teachers chose one model to be purchased for use in their classrooms.
**Week 6: Immunotherapy and Vaccines**	Teachers were introduced to new advances in Immunotherapy, what makes them work, and how cancer vaccines work. Teachers used the example of HPV vaccination to talk to their students about cancer prevention and encourage getting vaccinated.
**Week 7: Tobacco Control**	Discussion of the risks of smoking and other tobacco use including the risks of new e-cigarettes. Tools for talking to students about cancer risk and prevention.
**Week 8: Working meeting**	Finalize curriculum for use in the teacher's classrooms. Teachers also developed lesson plans for cancer prevention to be used in their classrooms.

**Table 2.** Demographics for Teachers and Schools at Which They Teach.

Demographic	Number	Percent
**Gender**		
Male	5	50%
Female	5	50%

**Race/Ethnicity**		
Black	4	40%
White	3	30%
Asian/Indian	3	30%

**Age**		
20-35 yrs	2	20%
36-50 yrs	2	20%
51-65 yrs	3	30%
> 65 yrs	3	30%

**Highest Degree**		
Bachelors	1	10%
Masters	8	80%
Doctorate	1	10%

**Program Participation**		
1 year*	4	40%
2 years	3	30%
3 years	3	30%

**School Location**		
Urban (CMSD)	8	80%
Suburban	2	20%

**School Level**		
Middle School	1	10%
High School	9	90%

**Subjects Taught** **		
Biology-Life Sciences	6	60%
Chemistry	2	20%
Environmental Sciences	2	20%
General Sciences	2	20%
Physics	1	10%

* Of the four teachers who have completed one year program participation, 3 have indicated their intent to return for at least a second year in 2022.

** Subjects taught total to greater than 100% since some teachers teach multiple subjects

**Table 3.** TTBC Participation.

	2018	2019	2020	2021	Total
New Teachers	4*	1	2**	3***	10
Returning Teachers (2ndyear)	0	3	2	1	6
Returning Teachers (3rd year)	0	0	1	2	3
Total Cohort	4	4	5	6	19

* 1 teacher returned in 2020.

** 1 teacher did not return due to personal reasons unrelated to the program.

*** All 3 new teachers in 2021 indicated they will return in 2022.

**Table 4.** Active Learning Strategies Incorporated Into Curriculum Design and Delivery.

• Case based learning (Srinivasan et al., 2007)
• Inquiry based learning (Edelson et al., 1999)
• Problem based learning (Wood, 2003)
• Personal subject matter relevance (Priniski et al., 2018)
• Social emotional learning (Weissberg et al., 2015)

**Table 5.** Key Observations from Program Evaluation and Teacher Feedback. Number of teachers expressing each opinion is provided in parentheses.

1.	Teachers lacked an understanding of the mission and activities of the NCI and Case CCC. (10 teachers)
2.	Teachers benefit from multiple years in the program. Both in increased knowledge about cancer and the structure and resources of the Case CCC. (6 teachers)
3.	Cohorts of 5-6 teachers are optimal. This cohort is big enough for teachers to work in groups with similar interests, but small enough to enable effective learning.
4.	Teachers preferred laboratory immersion with student pairs. Teachers could gauge the effectiveness of mentorship and educational needs of students. (10 teachers)
5.	Teachers often requested outreach to their schools from Case CCC Faculty in the year following their participation. (8 teachers)
6.	Teachers actively sought enhanced roles in the Case CCC. Including being active in the YES program curriculum and as community members of the Case CCC. (3 teachers)

## ABBREVIATIONS

Case CCC: Case Comprehensive Cancer Center
CMSD: Cleveland Metropolitan School District
CWRU: Case Western Reserve University
LTBC: Learn To Beat Cancer
NCI: National Cancer Institute
RTBC: Research To Beat Cancer
SEO: Scientific Enrichment Opportunity
SOM: School of Medicine
STEM: Science, Technology, Engineering and Math
TTBC: Teach To Beat Cancer
YES: Youth Enjoy Science
